# Heat Stress Alleviation by Exogenous Calcium in the Orchid *Dendrobium nobile* Lindl: A Biochemical and Transcriptomic Analysis

**DOI:** 10.3390/ijms241914692

**Published:** 2023-09-28

**Authors:** Yijun Fan, Jie Ma, Yuanyuan Liu, Xueyan Tan, Xuebing Li, Erya Xu, Linlong Xu, Aoxue Luo

**Affiliations:** Department of Landscape Plants, Sichuan Agricultural University, Chengdu 611130, China

**Keywords:** *Dendrobium nobile*, heat stress, exogenous calcium, transcriptome, gene expression

## Abstract

The growth of *Dendrobium nobile* is sensitive to heat stress. To find an effective method for enhancing heat tolerance, this study investigated the relieving effect of exogenous calcium at different concentrations (0 mmol/L, 5 mmol/L, 10 mmol/L, 15 mmol/L, 20 mmol/L CaCl_2_) on heat stress in *D. nobile*. Principal component analysis was used to screen the optimal exogenous calcium concentration, and transcriptome analysis was used to reveal its possible heat tolerance mechanism. The results showed that compared with the T0, a 10 mmol/L calcium treatment: increased the average leaf length, leaf width, plant height, and fresh matter accumulation of *D. nobile* by 76%, 103.39%, 12.97%, and 12.24%, respectively (*p* < 0.05); significantly increased chlorophyll a (Chla), chlorophyll b (Chlb), carotenoids(Car), ascorbic acid (ASA), glutathione (GSH), and flavonoids by 15.72%, 8.54%, 11.88%, 52.17%, 31.54%, and 36.12%, respectively; and effectively enhanced the enzyme activity of the antioxidant system, increasing superoxide dismutase (SOD), peroxidase (POD), and catalase (CAT) by 1.38, 1.61, and 2.16 times, respectively (*p* < 0.05); At the same time, the treatment can effectively reduce the yellow leaf rate and defoliation rate of *D. nobile* under heat stress. The principal component analysis method and membership function were used to calculate the D value to rank the relief effects of each calcium treatment group, and the results also showed that 10 mmol/L CaCl_2_ had the best relief effect. Transcriptomics testing identified 7013 differentially expressed genes, of which 2719 were upregulated, and 294 were downregulated. Among them, genes such as *HSPA1s*, *HSP90A*, *HSPBP1*, *ATG8*, *COMT*, *REF1*, *E1.11.1.7*, along with transcription factors such as MYB, bHLH, WRKY, and NAC, formed the network of tolerance to heat stress in *D. nobile*. This study provides new insights for improving the cultivation techniques of *D. nobile*.

## 1. Introduction

*Dendrobium nobile* Lindl. is a plant of the *Dendrobium* genus in the Orchidaceae family. It prefers cool and humid environments with a growth temperature of 18 °C to 30 °C. It is mainly distributed in subtropical areas such as Chishui in Guizhou and Hejiang in Sichuan, China [1]. Due to its beautiful flower color, abundant flower quantity, and beautiful form, *D. nobile* has high ornamental value. In addition, *D. nobile* is rich in active compounds such as polysaccharides [2], polyphenols [3], flavonoids [4], and alkaloids [5]. These compounds have hypoglycemic [6], anti-fatigue [7], immune regulation [8], anti-tumor [9], and antioxidant [10] functions. Under natural conditions, it is difficult for *D. nobile* to reproduce. Due to long-term destructive excavation and environmental damage, wild *D. nobile* populations are on the brink of extinction and the species has been listed as a second-level protected plant in China [11]. The artificial propagation and cultivation of *D. nobile* is the main means to meet market demand, and it is also of great significance for protecting wild *D. nobile* resources. As the main producing area of *D. nobile*, Chishui in Guizhou and Hejiang in Sichuan have an average temperature of 37 °C from July to September in summer [12]. High temperatures severely restrict the growth of *D. nobile*, reducing plant yield and medicinal quality. At the same time, global warming is unstoppable. According to the Intergovernmental Panel on Climate Change (IPCC) assessment, global temperatures will rise by 0.3–4.8 °C by the end of this century [13], and extreme high temperatures will become more frequent, posing greater challenges to agriculture. Summer high temperatures have become one of the main factors limiting the development of the Dendrobium industry, and this influencing factor will become increasingly severe. Therefore, finding effective ways to alleviate stress is an urgent problem to be solved in the cultivation and production of *D. nobile*.

Enhancing heat tolerance by adding exogenous substances is one of the most effective ways of solving the problem of plant susceptibility to high temperatures. Spraying chemical reagents, such as plant hormones or trace elements, can effectively reduce high-temperature damage [14]. Ca^2+^, as one of the essential nutrients for plant growth, plays an important role in signal transduction. The regulatory mechanism of Ca^2+^ on abiotic stress is mainly reflected in the regulation of the antioxidant system to eliminate reactive oxygen species, thereby protecting cell membrane integrity [15]. Ghosh et al. [16] studied the potential of calcium signaling and its transport mechanisms in plant stress tolerance and showed that an increase in Ca^2+^ concentration can activate stress-dependent kinases, thereby regulating stress response genes, thus having a mitigating effect on abiotic stresses such as drought, heat, heavy metals, and salt. Liang et al. [17] found that applying exogenous calcium can improve the antioxidant enzyme activity of rice leaves within a certain range, thereby alleviating the growth inhibition of rice seedlings under simulated acid rain environments. In addition, in recent years, numerous studies have shown that applying exogenous calcium may alleviate plant heat damage. Tan et al. [18] found that the external application of CaCl_2_ can reduce the inhibitory effect of heat stress on the net photosynthetic rate of tobacco, increase antioxidant enzyme activity, and reduce the content of superoxide anion free radicals and H_2_O_2_ in tobacco leaves under high temperatures. Calcium treatment can also enhance the binding of CaM (calmodulin) to the cytoplasm, resulting in extremely high plant heat tolerance. However, some studies have shown that the excessive application of exogenous calcium may cause damage to plants. This has been confirmed in studies on abiotic stress in *Gleditsia sinensis lam* [19] and *Sophora viciifolia* [20]. Therefore, the effectiveness of exogenous calcium in alleviating plant heat damage depends on various factors, such as plant species and calcium concentration. The present study induced heat stress in *D. nobile* using an artificial intelligence climate box and measured relevant indicators to explore the changes in appearance, morphology, physiology, and biochemistry under high temperatures. Exogenous calcium was used as a mitigating agent to explore its role in alleviating high-temperature stress, and the optimal concentration was identified. At the same time, transcriptomics techniques were used to analyze the gene expression in response to heat stress under treatment with exogenous calcium. This study was aimed at improving the cultivation techniques of *D. nobile* under high temperatures and, more generally, at exploring heat tolerance mechanisms in plants, and providing theoretical support for improving the heat tolerance and cultivation techniques of *D. nobile*.

## 2. Results

### 2.1. Effect of CaCl_2_ on the Growth of D. nobile under Heat Stress

As shown in Table 1, compared with the controls, the growth and development of *D. nobile* seedlings treated with heat were affected by varying degrees. The leaf length, leaf width, and plant height of *D. nobile* first increased, and then decreased, with increases in the CaCl_2_ concentration, reaching a maximum value in the T2 treatment and a minimum value in the T4 treatment. Compared with T0 (heat stress and no exogenous calcium), T1, T2, and T4 showed significant differences in leaf length (*p* < 0.05), while T2, T3, and T4 showed significant differences in leaf width and plant height (*p* < 0.05). After heat stress, the leaf length, leaf width, and plant height of *D. nobile* increased by 76%, 103.39%, and 12.97%, respectively, under the T2 treatment compared to the T0, and decreased by 76%, 54.24%, and 32.97% respectively under the T4 treatment compared to the T0. In summary, the T2 treatment had the best effect and, to some extent, alleviated the damage caused by heat to the growth and development of *D. nobile*.

From Table 1, it can be seen that the fresh weight of the underground and aboveground parts of the heat-stressed treatment group significantly decreased compared to the CK. Compared with T0 (heat-stressed plants with no exogenous calcium treatment), all parameters indicated a significant improvement in plant health associated with the calcium treatment, with a peak at T2, followed by a marked deterioration in T3 and T4. Compared with T0, the fresh root weight of *D. nobile* treated with T1 showed no significant difference (*p* > 0.05), while the other treatment groups showed significant differences (*p* < 0.05). The fresh root weight of T2 increased by 25.90% compared to that of the T0, while the fresh root weight of T4 decreased by 13.67% compared to that of the T0. The fresh stem weight of the T1, T2, T3, and T4 treatment groups showed significant differences compared to that of the T0 (*p* < 0.05). The fresh stem weight of T2 increased by 12.24% compared to that of the T0, while the fresh stem weight of T4 decreased by 33.88% compared to that of the T0. In terms of fresh leaf weight, there was a significant difference (*p* < 0.05) between each heat treatment group and the T0, with T2 increasing by 55.92% compared to that of the T0, and T4 increasing by 11.99% compared to that of the T0. The differences in root dry weight between T2, T3, T4 and T0 were significant (*p* < 0.05). In terms of stem dry weight, except for T1 and T0, there were significant differences between the other treatment groups and T0 (*p* < 0.05).

Except for the CK group, the yellow leaf rate and defoliation rate of the other treatment groups showed a trend of first decreasing and then increasing with the increase of CaCl_2_ concentration, and all showed significant differences from CK (*p* < 0.05). The yellow leaf rate and defoliation rate of the T1, T2, T3, and T4 treatment groups were significantly different from those of the T0 (*p* < 0.05). Among them, the T2 had the lowest yellow leaf rate and defoliation rate, which were 30.66% and 11.45% lower than those in the T0 treatment group, respectively. Compared with CK, the RWC of all heat treatment groups were significantly reduced (*p* < 0.05), with the T4 treatment group showing the lowest RWC, with a decrease of 53.12% and 29.36% compared to the CK and T0, respectively (*p* < 0.05). The RWC of T2 increased by 40.64%, 9.60%, 16.84%, and 99.10% compared to those of the T0, T1, T3, and T4, respectively (*p* < 0.05). It can be seen that the appropriate concentration of CaCl_2_ can effectively slow down the water loss of *D. nobile* leaves under heat stress, but also that high concentrations are clearly detrimental to the plant.

### 2.2. Effect of CaCl_2_ on the Antioxidant System of D. nobile under Heat Stress

**Figure 1:**
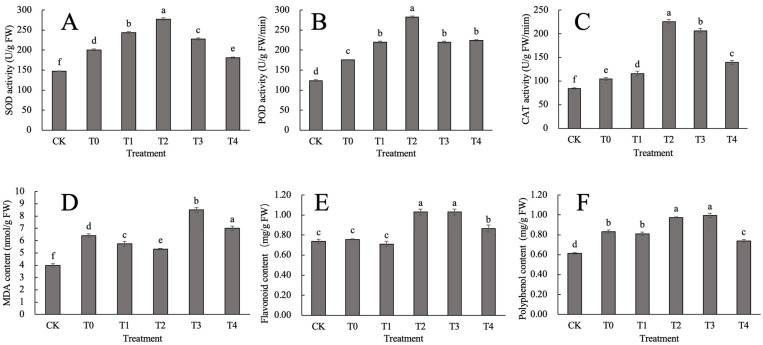
Effect of CaCl_2_ on the antioxidant system of *D. nobile* under heat stress: (**A**) SOD activity; (**B**) POD activity; (**C**) CAT activity; (**D**) MDA content; (**E**) flavonoids content; and (**F**) polyphenol content of *D. nobile* under heat stress. Different lowercase letters indicate significant differences among the treatments (*p* < 0.05).

High temperatures can cause the excessive production of reactive oxygen species within cells, thereby causing damage to plants. In order to investigate whether CaCl_2_ can alleviate the damage of *D. nobile* caused by heat stress through activating the antioxidant system, this study analyzed the antioxidant enzyme activity and antioxidant substances of *D. nobile*. The results, as shown in Figure 1, indicate that the SOD, POD and CAT activity of different concentrations of CaCl_2_ treatment groups showed an overall trend of first increasing, and then decreasing, under heat stress. Compared with the CK group, the SOD activity of each heat treatment group was significantly increased (*p* < 0.05). The SOD activity of *D. nobile* leaves under heat treatment reached its peak in the T2 concentration, which was 1.38 times higher than that of the T0 (*p* < 0.05) (Figure 1A). Meanwhile, the POD activity in the T2 treatment was 1.61 times higher than that seen in the T0 (*p* < 0.05). The POD activity in the T1, T3, and T4 treatments was significantly lower than that in the T2 treatment (*p* < 0.05) (Figure 1B). CAT activity was also the highest in the T2 treatment (Figure 1C). In summary, CaCl_2_ can activate the antioxidant enzyme activity of *D. nobile* to improve its heat tolerance, with the T2 concentration having the best effect.

From Figure 1D, it can be seen that, under heat stress, the malondialdehyde (MDA) content in the leaves of *D. nobile* significantly increased compared to that in the CK group (*p* < 0.05). The specific trend of change is as follows: with an increase in the CaCl_2_ concentration applied, MDA first decreased, then increased, and finally decreased. The MDA content in the T1 and T2 treatment groups significantly decreased compared to that in the T0 treatment (*p* < 0.05), with decreases of 9.65% and 17.66%, respectively. The T3 and T4 treatment groups showed a significant increase compared to that in the T0 (*p* < 0.05), with increases of 32.73% and 9.14%, respectively. It can be seen that the T2 treatment group was the most effective at reducing the accumulation of MDA, a membrane lipid peroxidation product, in the leaves of *D. nobile*.

The flavonoid content in the stems of *D. nobile* treated with different concentrations of CaCl_2_ after heat stress, compared to the CK group, first decreased, then increased, and eventually decreased again (Figure 1E). The T2, T3, and T4 treatments increased by 39.82%, 39.82%, and 17.19% compared to the CK group, respectively (*p* < 0.05). Compared with T0, the T2 and T3 treatments showed an increase of 36.12% (*p* < 0.05). The T4 treatment showed an increase in flavonoid content of 14.10% compared to that in the T0 treatment (*p* < 0.05). The trend of changes in polyphenol content was basically consistent with the trend observed in flavonoids (Figure 1F). The above results indicate that the T2 and T3 treatments can play an effective role in promoting the accumulation of flavonoids and polyphenols in the stems of *D. nobile* under heat stress.

### 2.3. Effect of CaCl_2_ on Photosynthetic Pigments in D. nobile under Heat Stress

Photosynthetic pigments have a central role in photosynthesis, and, quite predictably, the ability of plants to synthesize organic matter under heat stress decreases with a decrease in photosynthetic pigment content. In Table 2, it can be seen that the Chla, Chlb, Chla+b, and Car contents of each treatment group under heat stress were significantly lower than those of the CK group (*p* < 0.05) and showed a trend of first increasing, and then decreasing, with the increases in the CaCl_2_ concentrations applied. Each photosynthetic pigment reached its peak in the T2 treatment group, with an increase of 15.72% (*p* < 0.05), 8.54% (*p* < 0.05), 33.50% (*p* < 0.05), and 11.88% (*p* < 0.05) compared to those in the T0 treatment group, respectively. In addition, the Chla, Chlb, and Car contents of each heat treatment group reached their lowest values in the T4 treatment group, which were significantly reduced by 16.35%, 7.32%, and 9.96% compared to those in the T0 treatment group (*p* < 0.05). The content of Chla+b was significantly increased in T2 relative to T0 (*p* < 0.05). The above indicates that spraying CaCl_2_ with the T2 concentration on the leaves of *D. nobile* elicits the highest photosynthetic pigment synthesis rate under heat stress.

### 2.4. Effect of CaCl_2_ on the Osmotic Regulation System of D. nobile under Heat Stress

**Figure 2:**
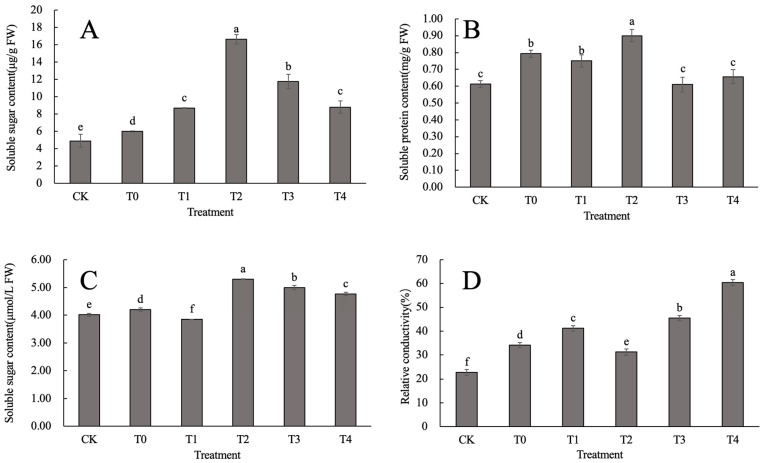
Effect of CaCl_2_ on the osmotic regulatory system of *D. nobile* under heat stress: (**A**) free Pro content; (**B**) SP; (**C**) SS; and (**D**) REC of *D. nobile* under heat stress. Different lowercase letters indicate significant differences among the treatments (*p* < 0.05).

The Pro contents in the calcium-treated groups were significantly higher than those in the CK and T0 groups (Figure 2A). The Pro content in the T2 treatment was 3.39 and 1.92 times higher than that in the CK and T0, respectively, and was significantly higher than that in T1, T3, and T4. SP is the main anti-heat osmotic regulator in plant cells and has a central role in the control of osmotic pressure in plant cells. The effect of foliar spraying of CaCl_2_ on the SP content of *D. nobile*, as shown in Figure 2B. The SP content of the T0, T1, and T2 treatment groups was significantly increased compared with that in the CK. Compared with T0, although there was a significant difference (*p* < 0.05) between T2, T3, and T4 and T0, only the T2 treatment had a significantly higher SP content than the T0, with an increase of 13.45% compared to the T0. Figure 2C shows that, except for the T1 treatment group, the SS content of all calcium-treated groups significantly increased compared to the CK: the T2, T3, and T4 treatments showed significant increases of 26.17%, 18.95%, and 11.40% (*p* < 0.05) compared that in the T0. It can be seen that the induction effect of CaCl_2_ in the T2 concentration on the SS content was the most significant, followed by the T3 treatment group. In summary, the T2 concentration of CaCl_2_ was the most effective at promoting an increase in the accumulation of Pro, SP, and SS contents in the leaves of *D. nobile* under heat stress; the use of this treatment can effectively alleviate the enormous pressure caused by heat stress on the osmotic regulatory system.

Heat stress significantly increased the REC value of *D. nobile* leaves (*p* < 0.05) (Figure 2D). Among the treatments, the REC value of T2 significantly decreased (*p* < 0.05), and was 8.53% lower than that of the T0 treatment. The REC value reached its highest value in the T4 treatment, which was 1.77 times higher than that in the T0 (*p* < 0.05). Although the REC values of the T1 and T3 treatments were significantly higher than those of the CK and T0, the amplitude of change was relatively small. From this it can be seen that the cell membrane of *D. nobile* may be damaged to some extent under heat stress; however, application of the T2 concentration of CaCl_2_ can alleviate tropical damage.

### 2.5. Effect of CaCl_2_ on the Ascorbic Acid Glutathione Cycle in D. nobile under Heat Stress

**Figure 3:**
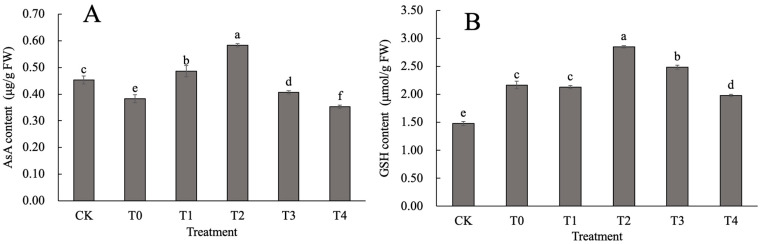
Effect of CaCl_2_ on the ascorbic acid glutathione cycle in *D. nobile* under heat stress: (**A**) ascorbic acid content; and (**B**) glutathione content of *D. nobile* under heat stress. Different lowercase letters indicate significant differences among the treatments (*p* < 0.05).

ASA is an important antioxidant in plants because it can eliminate free radicals in enzyme molecular structures and alleviate membrane damage. From Figure 3A, it can be seen that, as the spraying concentrations increase, the ASA content in each heat treatment group first increases and then decreases. Only the T2 and T1 treatments show a significant increase in ASA content, while T0, T3, and T4 have a significantly lower ASA content than that in the CK group. Compared with the T0 treatment, only the T4 treatment showed a significant decrease, while the ASA content in the other heat groups was significantly higher than that in the T0 (*p* < 0.05). The ASA content in the T1, T2, and T3 treatments increased by 26.96%, 52.17%, and 6.09%, respectively, compared to that in the T0. It can be seen that applying the T1 and T2 concentrations of CaCl_2_ under heat stress had a better promoting effect on the ASA content in the leaves of *D. nobile*, with the T2 treatment having a better effect than the T1 treatment.

From Figure 3B, it can be seen that the trend of changes in the GSH leaf content under heat stress is generally consistent with that of the ASA. Compared with the CK, the GSH content in each heat treatment group was significantly increased (*p* < 0.05), while the T2 showed an increase of 92.57% compared to that of the CK. T3 showed an increase of 68.24% compared to that of the CK. Compared with the T0, T1 showed no significant difference (*p* > 0.05). The GSH leaf content in T2 and T3 significantly increased, by 31.54% and 14.92% (*p* < 0.05), respectively. GSH leaf content in T4 significantly decreased, by 8.62% (*p* < 0.05). Therefore, the exogenous application of CaCl_2_ had, to some extent, slowed down the decrease in GSH content under heat stress, thus probably contributing towards alleviating damage to the leaves of *D. nobile*. Among the groups, the T2 treatment group had the most significant effect.

### 2.6. Comprehensive Evaluation

#### 2.6.1. Correlation Analysis

**Figure 4:**
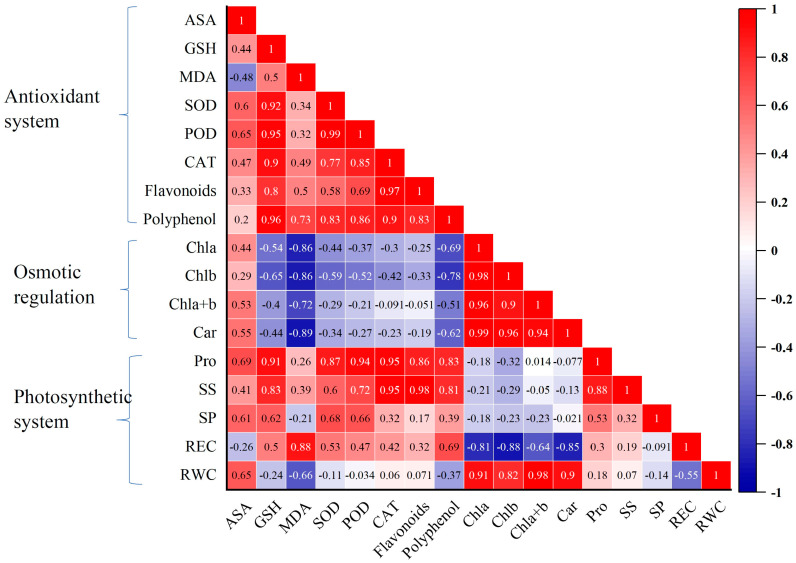
Correlation analysis of physiology and biochemistry indicators.

The correlation between various physiological and secondary metabolic indicators in the responses of *D. nobile* to different concentrations of CaCl_2_ under heat stress is shown in Figure 4. It can be seen that MDA was significantly negatively correlated with Chla and Car (*p* < 0.05), and extremely significantly negatively correlated with Chlb, SP, and ASA (*p* < 0.01). Chla was significantly positively correlated with Chlb, Chla+b, Car, SOD, SP, ASA, and GSH (*p* < 0.01), and was significantly positively correlated with Pro and polyphenols (*p* < 0.05). Chlb was significantly positively correlated with Chla+b, Car, SOD, SP, ASA, and GSH (*p* < 0.01), and was significantly positively correlated with Pro (*p* < 0.05). Chla+b was significantly positively correlated with Car, SOD, CAT, POD, Pro, ASA, and GSH (*p* < 0.01). SOD was significantly positively correlated with CAT and SP (*p* < 0.05), and extremely significantly positively correlated with POD, Pro, ASA, GHS, and polyphenols (*p* < 0.01). POD was significantly positively correlated with CAT, Pro, SS, ASA, GSH, and xanthone (*p* < 0.01). CAT showed a highly significant positive correlation with Pro, SS, GSH, flavonoids, and polyphenols (*p* < 0.01), and a significant positive correlation with ASA (*p* < 0.05). ASA was significantly positively correlated with polyphenols (*p* < 0.05), and extremely significantly positively correlated with GSH (*p* < 0.01). GSH had a highly significant positive correlation with flavonoids and polyphenols (*p* < 0.01). There was a highly significant positive correlation between flavonoids and polyphenols (*p* < 0.01).

#### 2.6.2. Principal Component Analysis

The responses of various physiological and biochemical indicators to exogenous calcium in *D. nobile* under heat stress varied, and the amplitude of changes for each individual indicator was not entirely consistent. The contribution of various indicators in enhancing the heat resistance of *D. nobile* with exogenous calcium is different. Therefore, using a single indicator to evaluate the relief ability of exogenous calcium in *D. nobile* under heat stress had limitations. To obtain better results, we evaluated the data comprehensively across multiple dimensions. Table S1 indicates that there was a certain correlation between most indicators. Therefore, in order to reduce information intersection and overlap, principal component analysis was used to obtain the heat tolerance coefficients of 17 individual indicators, as shown in Table S2. According to the principle of eigenvalues greater than 1, the original 17S2 individual indicators were converted into three new and independent comprehensive indicators. The contribution rates of these three comprehensive indicators were 62.649%, 22.965%, and 8.146%, respectively, representing 93.76% of the information in the original 17 indicators.

The characteristic value of the first principal component was 10.65, and the main contribution indicators included RWC, Chla, Chlb, Car, SOD, Pro, ASA, GSH, which reflects the ability of exogenous calcium to maintain the water, photosynthesis, osmotic regulation, and antioxidant capacity of *D. nobile* plants under heat stress. The characteristic value of the second principal component was 3.904, and the main contribution indicators included CAT, SS, SP, and flavonoids, mainly reflecting their osmoregulation ability and the accumulation of some effective components. The characteristic value of the third principal component was 1.385, and the main contribution indicators included REC, MDA, Chla+b, POD, and polyphenols, mainly reflecting the ability to reduce damage to cell membranes, free radical scavenging, and the accumulation of effective components.

According to the principal component analysis, the eigenvectors in Table S1 were used as coefficients for each comprehensive indicator to calculate the comprehensive score values of the first, second, and third principal components, represented by Y1, Y2, and Y3, respectively (Table 3). The calculation formula was as follows:

Y1 = 0.271 × X1 − 0.212 × X2 − 0.140 × X3 + 0.268 × X4 + 0.255 × X5 + 0.262 × X6 + 0.279 × X7 + 0.297 × X8 + 0.243 × X9 + 0.223 × X10 + 0.268 × X11 + 0.149 × X12 + 0.202 × X13 + 0.290 × X14 + 0.284 × X15 + 0.170 × X16 + 0.232 × X17

Y2 = −0.105 × X1 + 0.251 × X2 + 0.357 × X3 − 0.197 × X4−0.219 × X5 + 0.029 × X6 − 0.192 × X7 − 0.069 × X8 + 0.160 × X9 + 0.345 × X10 + 0.221 × X11 + 0.404 × X12 − 0.263 × X13 − 0.116 × X14 + 0.144 × X15 + 0.411 × X16 + 0.202 × X17

Y3 = −0.250 × X1 + 0.357 × X2 − 0.418X3−0.195 × X4 − 0.022 × X5 + 0.291 × X6 − 0.094 × X7 − 0.024 × X8 + 0.436 × X9 + 0.014 × X10 + 0.176 × X11 + 0.125 × X12 + 0.174 × X13 + 0.155 × X14 − 0.139 × X15 + 0.050 × X16 − 0.436 × X17

#### 2.6.3. Comprehensive Analysis of Membership Functions

Based on the results and expressions of the principal component analysis, the comprehensive indicator value Y (X) and corresponding membership function value U (x) for each treatment were calculated, as shown in Table 3. For the comprehensive indicator Y1, the U1 value of the T2 treatment was the highest, indicating that the T2 treatment exhibited the strongest heat tolerance on this comprehensive indicator, while the membership function value of T4 treatment was the lowest, indicating that the T4 treatment had the worst heat tolerance on Y1. According to the formula, the weights of the three comprehensive indicators were 0.667, 0.244, and 0.087, respectively. Finally, combined with the weight values, the comprehensive evaluation value D was calculated. The larger the D value, the stronger the heat tolerance. According to the D value, the relieving effect of different concentrations of exogenous calcium on *D. nobile* under heat stress was ranked as T2 > T3 > T1 > T4 > T0, indicating that, in this experiment, the concentration of CaCl_2_ at T2 was the most effective in alleviating heat stress damage in *D. nobile* plants.

### 2.7. Transcriptome Sequencing Quality Analysis and Differential Gene Screening

**Figure 5:**
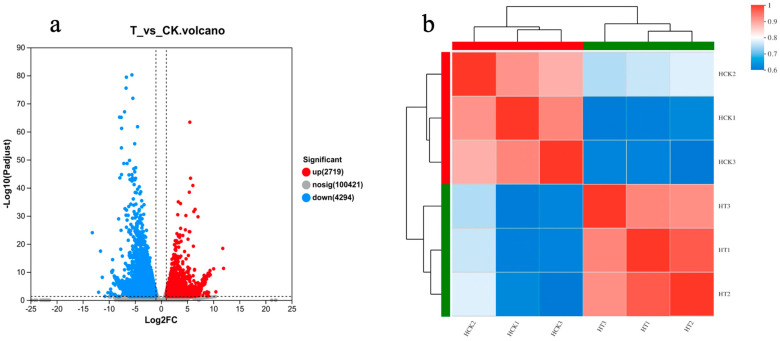
Differential gene screening results: (**a**) volcano plot; and (**b**) cluster analysis.

The transcriptome analysis of six samples was completed in this experiment, and a total of 39.71Gb of clean data was obtained. The clean data of each sample were always above 6.44 Gb, with a Q30 base percentage of over 92.86% and a GC content of over 45%. This indicates that the sequencing quality was good and could be further analyzed (Table S2). Using Trinity to perform de novo assembly on all sample clean data and optimize the assembly results, the assembled unigenes were 107,434, with 168,455 transitions, an average length of 708.51 bp, and an N50 length of 1194 bp. The standard for screening differential genes this time was *p* < 0.05. Compared with the control group, a total of 7013 differentially expressed genes (DEGs) were obtained after screening, of which 2719 were upregulated and 4294 were downregulated (Figure 5).

### 2.8. GO Functional Enrichment of DGEs

**Figure 6:**
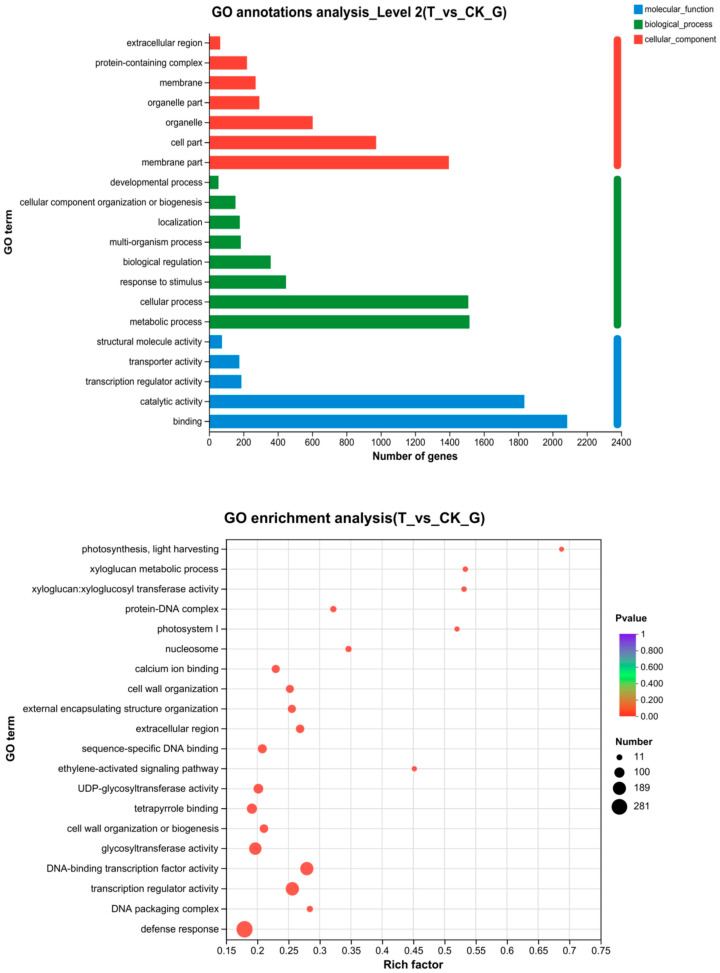
GO functional enrichment analysis of differentially expressed genes.

GO analysis [21] provides a functional annotation of genes within three categories, namely, the cellular components, biological processes, and molecular functions. GO analysis was performed on 4294 DEGs between the treatment and the CK, and a total of 19 biological processes, 12 cellular components, and 15 molecular functions were enriched (Figure 6). The GO functional annotations of cellular components mainly included the protein-containing complex, membrane, organelle part, organelle, cell part, and membrane part. The biological processes mainly included the developmental process, cellular component organization or biogenesis, localization, multi-organism process, biological regulation, response to stimulus, cellular, and metabolic processes. Molecular function mainly included structural molecule activity, transporter activity, transcription regulator activity, catalytic activity, and binding.

### 2.9. KEGG Analysis of DEGs

**Figure 7:**
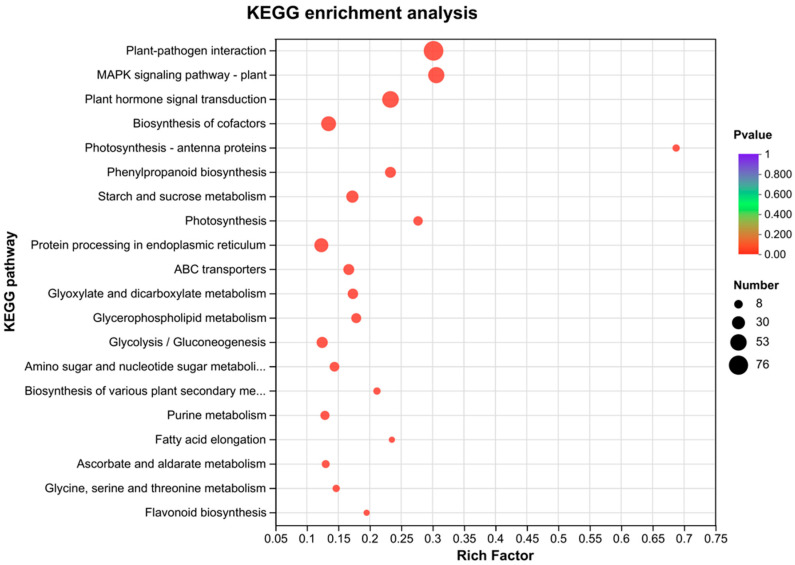
KEGG functional enrichment analysis of differential genes.

Results for the KEGG [22] annotation for the metabolic pathway enrichment analysis showed that 4294 DEGs were involved in 122 KEGG metabolic pathways. The top 20 metabolic pathways included: plant pathway interaction (76 DEGs); MAPK signaling pathway-plant (52 DEGs); plant hormone signal transduction (55 DEGs); biosynthesis of cofactors (24 DEGs); photosynthesis antenna proteins (44 DEGs); phenylpropanoid biosynthesis (11 DEGs); starch and sucrose metabolism (30 DEGs); photosynthesis (18 DEGs); protein processing in endoplasmic reticulum (39 DEGs); ABC transporters (24 DEGs); glyoxylate and dicarboxylate metabolism (22 DEGs); glycerophospholipid metabolism (20 DEGs); glycolysis/gluconeogenesis (25 DEGs); amino sugar and nucleotide sugar metabolism (19 DEGs); biosynthesis of various plant secondary metabolites (11 DEGs); purine metabolism (17 DEGs); fatty acid elongation (8 DEGs); ascorbate and aldarate metabolism (8 DEGs); glycine, serine and threonine metabolism (13 DEGs); and flavonoid biosynthesis (11 DEGs) (Figure 7).

### 2.10. Key Gene Analysis

DN3260(*HSPA1s*), DN45157(*IRE1A*), DN4329(*EIF2AK4*), DN55311(*HSP90A*), DN1482(*HSPBP1*), DN9077(*SEC61A*), DN14663(*SKP1*), DN5970(*SKP1*) genes in protein processing in the endoplasmic reticulum were significantly upregulated.

DN2523(*ATG8*), DN14276(*ATG8*), DN3173(*ATG8*) genes in the autophagy pathway were significantly upregulated.

DN34241(*OXI1*), DN3317(*copA*), DN440 (*MYC2*), DN15471(*CAT*), DN3164(*SNRK2*), DN3350(*EIN3*), DN37771(*PYL*), DN19125(*PYL5*), DN26116(*EPF1*), DN245(*CAT*), DN3317(*copA*) genes in the MAPK signaling pathway were significantly upregulated.

**Figure 8:**
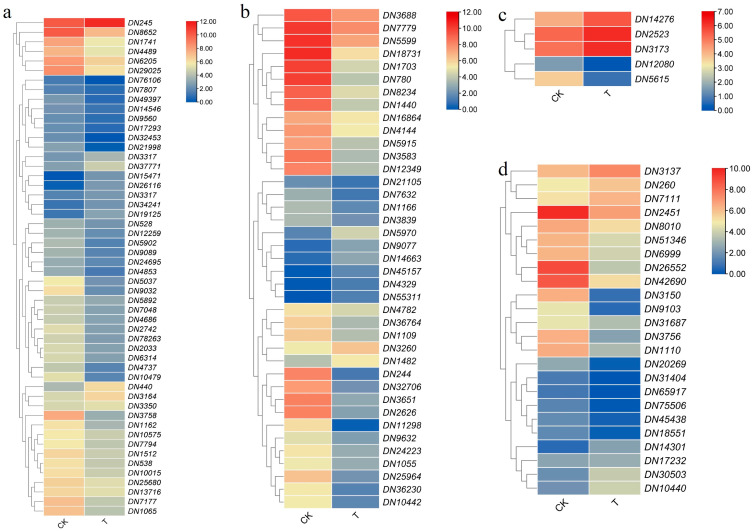
Analysis of key differentially expressed genes: (**a**) MAPK signaling pathway; (**b**) protein processing in endoplasmic reticulum; (**c**) autophagy; and (**d**) phenylpropanoid biosynthesis.

DN260(*E1.11.1.7*), DN14301(*E1.11.1.7*), DN30503(*E1.11.1.7*), DN7111(*COMT*), DN3137(*REF1*), DN10440(*E1.11.1.7*) in the phenylpropanoid biosynthesis were significantly upregulated (Figure 8).

### 2.11. Transcription Factor Analysis

**Figure 9:**
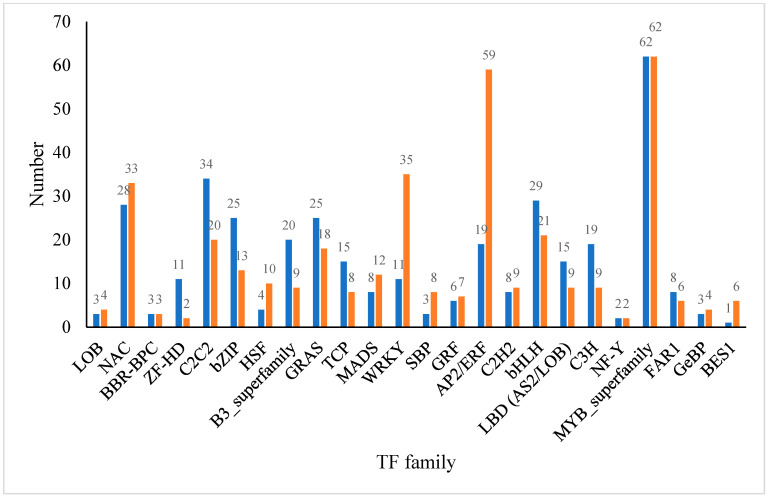
Transcription factor analysis family analysis.

As shown in Figure 9, a summary analysis was conducted on the transcription factor family genes. Compared with CK, the treatment group had a total of 751 differentially expressed transcription factor genes, among which the AP2/ERF-ERF, MYB_superfamily and C2H2 families had the most genes, with 78, 124, and 54 genes, respectively. The differential expression of transcription factor genes is located in the TOP 20 with: the MYB_superfamily(Up44, Down35); AP2/ERF(Up19, Down59); NAC(Up28, Down33); C2C2(Up34, Down20); bHLH(Up29, Down21); WRKY(Up11, Down35); GRAS(Up25, Down18); bZIP(Up25, Down13); B3_superfamily(Up20, Down9); C3H(Up19, Down9); LBD(Up15, Down9); TCP(Up15, Down8); MADS(Up8, Down12); C2H2(Up8, Down9); HSF(Up4 Down10); FAR1(Up8, Down6); ZF-HD(Up11, Down13); GRF(Up6, Down7); SBP(Up3, Down8); and LOB(Up3, Down7). It is speculated that they play an important role in alleviating the response of *D. nobile* to heat stress with the addition of calcium.

### 2.12. qRT-PCR Analysis of Differential Gene Expression Levels

To further verify the reliability of transcriptome sequencing results, nine differentially expressed genes (*DN3260*, *DN45157*, *DN14663*, *DN2523*, *DN3317*, *DN440*, *DN3350*, *DN19125*, *DN7111*) were randomly selected for qRT-PCR validation (Figure S1). The results showed that the relative expression levels of the nine detected differential genes were consistent with the trend of the transcriptome sequencing results, indicating that the transcriptome sequencing results have high reliability.

## 3. Discussion

### 3.1. Effects of Exogenous Calcium on Physiological and Biochemical Characteristics of D. nobile under Heat Stress

Heat stress can cause a series of adverse effects on plant growth and development, such as leaf chlorosis, wilting, shedding, and even plant death. In this experiment, after 15 days of 40 °C stress, the leaf length, leaf width, plant height, and the fresh and dry weight of roots, stems, and leaves of each treatment group were lower than CK, indicating that heat stress had an adverse effect on the growth of *D. nobile*. However, under heat treatment, T1 and T2 had a promoting effect on the growth, development, and biomass accumulation compared to T0, indicating that spraying appropriate concentrations of CaCl_2_ can effectively reduce the damage of heat stress to *D. nobile*, similar to results obtained in the research of Lu et al. [23]. Continuous heat stress can disrupt the relative balance of ROS in plants, leading to membrane lipid peroxidation in plant cells and an increase in MDA and REC values [24]. In this experiment, the REC and MDA of each heat treatment were significantly higher than CK, indicating that even with CaCl_2_ treatment, heat damage to *D. nobile* plants cannot be completely avoided. Heat stress has a serious impact on the chloroplast structure of plants and adding exogenous calcium can improve the stability of the plant cell membrane and chloroplast membrane structure, thereby reducing the loss of photosynthetic pigments [25]. In this experiment, the Chla, Chlb, Chla+b, and Car contents of each group under heat treatment were significantly reduced compared to the room temperature control group CK. Compared to the hot control T0, the T1 and T2 treatment groups were significantly increased, indicating that the appropriate concentration of CaCl_2_ spray maintained the synthesis of photosynthetic pigments. This is consistent with the research results of Hu et al. [26]. Interestingly, there was no significant difference in photosynthetic pigment content between the T3 treatment and T0 treatment, while the content of photosynthetic pigments decreased significantly under the T4 treatment. This may be due to the ion toxicity caused by high concentrations of Ca^2+^, which inhibited plant photosynthesis. Continuous heat stress can cause the accumulation of reactive oxygen species in plants to exceed the tolerance threshold, leading to the inactivation and denaturation of antioxidant enzymes. In recent years, researchers have attempted to increase plant antioxidant enzyme activity to resist heat damage by adding different exogenous substances [27]. In this experiment, we attempted to improve the tolerance of *D. nobile* to heat stress by applying different concentrations of CaCl_2_. The results showed that T2 had the highest increase in SOD, POD, and CAT activities, indicating that this concentration can effectively induce an increase in protective enzyme activity in *D. nobile*.

When plants are under abiotic stress, they regulate the osmotic pressure of plant cells by increasing the content of osmoregulation substances. SS, SP, and Pro are important osmoregulation substances. In the present study, compared with CK and T0, the Pro content in each heat treatment group significantly increased, and the overall trend showed an increase followed by a decrease. This may be due to the fact that the plant itself would resist stress by inducing an increase in Pro under heat stress. In addition, different concentrations of CaCl_2_ treatment may promote the participation of Ca signaling systems in this induction process, leading to an increase in Pro content. Meanwhile, compared with T0, the T2 treatment significantly increased SP and SS content, showing a positive relieving effect. The results of the study indicate that only suitable concentrations of exogenous substances can improve the heat tolerance of plants, and that high concentrations of exogenous substances may weaken this relief effect. Research on honeysuckle [28] has also confirmed this.

The accumulation of flavonoids and polyphenols can improve the antioxidant capacity of plants. In this experiment, the flavonoid and polyphenol contents of *D. nobile* under different heat treatment groups were generally increased by varying degrees and showed a trend of first increasing and then decreasing, which is consistent with the research results obtained by Xia et al. [29]. In the present study, the ASA and GSH contents of each heat treatment group showed a trend as flavonoids, but the specific changes were different. Among them, the ASA content of the T1, T2, and T3 treatments was significantly higher than that of the T0 treatment group, with the T2 treatment having the largest increase. The GSH content was significantly increased in the T2 and T3 treatments, with the largest increase in the T2 treatment. This indicates that the plant itself enhances stress tolerance by increasing GSH activity under thermal stress. However, spraying CaCl_2_ helps the *D. nobile* plant to better adapt to the thermal environment, thereby reducing membrane lipid peroxidation and improving plant heat tolerance, similar to the results obtained in research by Du et al. [30].

### 3.2. Effect of Exogenous Calcium on Gene Expression in D. nobile under Heat Stress

The perception and transmission of high-temperature stress signals in plants include multiple pathways, among which protein kinases mainly serve to transmit extracellular high-temperature stimuli to cells, mediating various cellular signaling network pathways to regulate gene expression and protein function, including mitogen-activated protein kinases (MAPK/MPKs), calcium-dependent protein kinases (CDPKs), signaling molecules, and plant hormones [31,32], ultimately enabling plants to adapt to environmental stress.

Heat shock proteins (HSPs) are present in almost all organisms and play a crucial role in plant survival under heat stress. HSPs can be divided into *HSP100*, *HSP90*, *HSP70*, *HSP60* and *HSP20*. *Hsp90s* is a widespread and highly conserved heat shock protein gene family that plays an important role in assisting protein folding and promoting cell signal transduction under heat stress [33]. *HSPBP* contains a tiny functional domain, which often appears in plant transcription factors containing GATA and has transcription factor characteristics. It is a nuclear localization site and DNA-binding region. In this study, protein genes such as *HSPA1s*, *HSP90A* and *HSPBP1* were significantly upregulated in the treatment group. Therefore, we can speculate that after adding exogenous calcium, the heat stress signal of *D. nobile* is sensed by *HSP90A*, and that *HSP90A* binds to *HSPBP1*, transferring the signal to *HSPBP1*. *HSPBP1* is continuously synthesized as a transcription factor, transmitting and amplifying cytoplasmic signals into the nucleus, and binding to the cis-acting element of stress response genes. Regulating the expression of these genes plays a role in the tolerance of *D. nobile* to heat stress.

High-temperature stress can induce the expression of autophagy genes and promote the accumulation of autophagosomes. Thus, autophagy is considered to be involved in plant responses to high-temperature stress [34]. *HSP* is also involved in the process of cell autophagy. Selective autophagy refers to the cellular autophagy mediated by specific molecular chaperones such as *HSC70* and HSP90, targeting organelles, macromolecules, protein complexes, toxic proteins, and invading pathogens that require clearance within cells. *ATG8* plays an important role in the process of autophagy. Studies in chili peppers have found that the silent expression of *CaATG8c* negatively regulates the heat and salt tolerance of chili peppers. However, the overexpression of *CaATG8c* in Arabidopsis shows strong sensitivity to heat and salt stress [35]. In this study, the *ATG8* gene in the treatment group was significantly upregulated.

**Figure 10:**
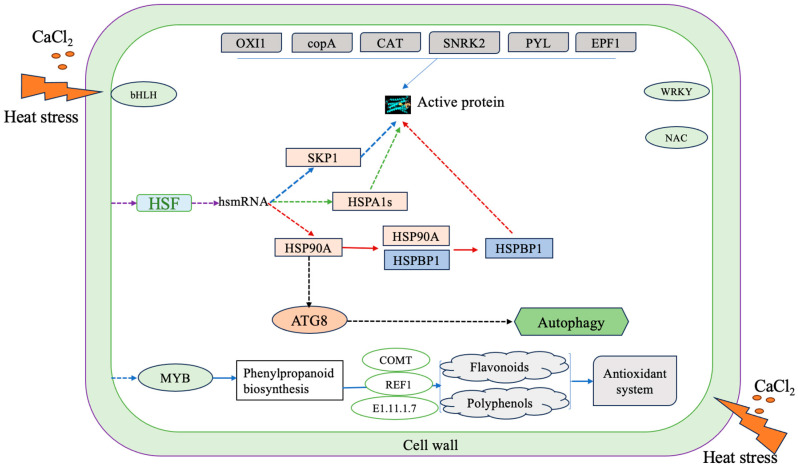
Effect of exogenous calcium on gene expression in *D. nobile* under heat stress.

Plants can induce and activate the MAPK pathway under different stress conditions, such as salt damage, high temperatures, and drought, continuously amplifying and transmitting signals [36]. Huang et al. [37] found in their research on rice MAPK that heat stress can quickly lead to a decrease in the expression of the *OsMAPK2* gene. Yelena et al. [38] found in their tobacco research that plants transfected with the *MPK1* gene can greatly improve heat tolerance. In this study, genes such as *OXI1*, *copA*, *MYC2*, *CAT*, *SNRK2*, *EIN3*, *PYL*, *PYL5*, and *EPF1* were significantly upregulated in the MAPK signaling pathway of the treatment group. Therefore, it can be inferred that these genes play an important role in the tolerance of *D. nobile* to heat stress with the addition of exogenous calcium (Figure 10).

### 3.3. The Role of Transcription Factors in Enhancing the Tolerance of D. nobile to Heat Stress by Exogenous Calcium

Hsfs (heat shock transcription factors) play an important role in plant heat tolerance, minimizing damage and ensuring dynamic cell balance through various responses to high-temperature stress. Han et al. [39] identified 23 members of the Hsf gene family in Dendrobium officinale that play key roles in different stress or plant hormone signaling pathways. In addition to Hsfs, other larger transcription factor families in plants are also involved in the response to high-temperature stress, such as bZIP, NF-Y, NAC, WRKY, and MYB transcription factor families [40]. For example, overexpression of the *AtMBFlc* gene in Arabidopsis can lead to an increase in the expression levels of *AtWRKY18*, *AtWRKY46*, *AtWRKY40* [41], and *AtWRKY33* [42], thereby improving their heat tolerance. The overexpression of AtNF-YC10 in Arabidopsis and rice can significantly improve the plant’s tolerance to high temperatures [43]. *MYB30* in Arabidopsis can regulate heat stress response by regulating calcium signaling [44]. Meanwhile, MYB transcription factors are involved in regulating the synthesis of flavonoids and floral aroma compounds in orchids [45]. In this study, four HSFs—29 bHLHs, 11 WRKYs, 28 NACs—and 44 MYB transcription factors were significantly upregulated in the treatment group. We speculate that these play an important role in alleviating the heat stress response of *D. nobile* with the addition of calcium (Figure 9).

## 4. Materials and Methods

### 4.1. Materials and Treatments

The annual *D. nobile* were purchased from Longquan Original Ecological Industry Industrial Co., Ltd. in Chishui City, China. The plants were placed in pots with a height of 13.5 cm and an inner diameter of 14.5 cm, with a maintenance substrate of wood chips and ceramic particles (1:2). The experiment was conducted at the experimental base of Sichuan Agricultural University in China, with all plants under conventional water and fertilizer management. The cultivation temperature was 20 °C, the photoperiod was 12 h/12 h, the relative humidity was 80%, and the light intensity was 3600 lx. Plants were watered every two days on average, with 100 mL of water per plant pot. After 15 days, plants (720) in good health and of a similar size were selected for the experiment.

In the previous study, 25 °C was used as the control to investigate the effects of 4 high-temperature treatments (30 °C, 35 °C, 40 °C, 45 °C) on the growth of *D. nobile*. The results showed that *D. nobile* exhibited good tolerance under treatments at 30 °C and 35 °C. When the temperature reached 40 °C, significant damage to the plants was observed, but after 14 days of cultivation at room temperature, the plants often recovered to the control level or approached the control. The 45 °C treatment caused the most severe damage to the plants, so that they could not recover even after 14 days of stress relief, indicating that the damage to the plants caused by this treatment was irreversible. Therefore, based on the results of the heat stress pre-experiment, CaCl_2_ (purchased from Chengdu Kelong Chemical Company, Chengdu, China) mitigation experiments were conducted at the same treatment temperatures and time periods that caused certain damage to plants but did not reach irreparable levels. The heat stress conditions were a day/night temperature of 40 °C/35 °C, and CK was a normal temperature control (25 °C/20 °C). CaCl_2_ solutions with concentrations of T0 (0 mmol/L), T1 (5 mmol/L), T2 (10 mmol/L), T3 (15 mmol/L), and T4 (20 mmol/L) were sprayed on the leaves of the plants once a day until the solution was ready to drip. The treatment lasted for 15 days, and relevant indicators were sampled and tested. A total of 120 seedlings were divided into 3 parallel groups for each treatment, with 40 seedlings in each group.

### 4.2. Detection of Growth Indicators

Three seedlings of *D. nobile* were randomly selected from each treatment and their morphological indicators were measured at fixed points using a five-point sampling method, with 3 replicates. A ruler (with an accuracy of 0.1 cm) was used to measure plant height (the distance from the root of the plant to the growth point). Leaf length, leaf width and leaf area were measured using a leaf area meter. The number of leaves was recorded, including the fallen leaves. All *D. nobile* plants (48 in each group) were collected, washed, and the surfaces were dried with paper. The fresh weights of the plant roots, stems, and leaves were measured. The plants were then placed in an oven for drying, and the dry weights of each treated root, stem, and leaf were weighed separately.

### 4.3. Determination of Physiological and Biochemical Indicators

The detection of relative conductivity (REC) was conducted according to the method of Liu et al. [46]. The thiobarbituric acid method [47] was used to determine the content of malondialdehyde (MDA). The methods of Fan et al. [48] were used to determine the contents of chlorophyll a, chlorophyll b, and carotenoids (Car) in the leaves of *D. nobile*, as well as the activities of SOD(EC 1.15.1.1), POD(EC 1.11.1.7) and CAT(EC 1.11.1.6). The contents of free proline (Pro), soluble protein (SP), ascorbic acid (ASA), and glutathione (GSH) were detected using the method of Vuleta et al. [49]. The soluble sugar (SS) content was determined using the phenol sulfuric acid method [50,51].

The total flavonoid content of Dendrobium was determined by the aluminum nitrate method [52]. An amount of 0.2 g of *D. nobile* powder was weighed and extracted with 70% ethanol at 60 °C for 3 h. A certain amount of the extraction solution was diluted, and 0.3 mL of 10% aluminum nitrate solution and 2 mL of 4% sodium hydroxide solution were added, respectively. After reacting for 10 min, the OD value was measured at wavelength of 510 using a spectrophotometer. The standard curve of the total flavonoids was constructed using rutin.

The polyphenol content was determined using the Folin–Ciocalteu (FC) method [52]. A stem sample (0.1 g) was accurately weighed and placed in a 100 mL Erlenmeyer flask. The sample was treated with 50 mL of 70% ethanol, shaken well for 4 h, and filtered. The filtrate was diluted to 100 mL, and then 1.0 mL of the extract was mixed with 0.3 mL of the FC reagent for 5 min. The sample was treated with 1.5 mL of saturated sodium carbonate solution and the volume was made up to 10 mL with water. The mixture obtained was incubated for 30 min in a 30 °C water bath. Absorbance was then measured at a 760 nm wavelength. A standard curve was constructed with gallic acid.

### 4.4. Transcriptome Sequencing

#### 4.4.1. Illumina Sequencing

Leaf samples of *D. nobile* were washed, wrapped in tin foil, and quickly plunged into liquid nitrogen. The total RNA was extracted from different treatment groups, and RNA samples that had undergone quality testing were sent to Shanghai Meiji Biological Information Technology Co., Ltd. (Shanghai, China) to construct a library using the Illumina HiSeq2100 platform. Internal Perl script processing was performed on the raw data obtained based on the sequencing platform, which included removing low-quality reads and reads containing more than 10% uncertain bases. Meanwhile, clean reads were evaluated based on Q20, Q30, and GC content. Trinity software(v2.4.0) was used to assemble high-quality reads [53].

#### 4.4.2. Differential Gene Enrichment Analysis

We compared the high-quality clean data obtained by removing connector sequences and low-quality reads from the raw data with the specified reference genome sequence to obtain mapped data for the gene expression analysis in the sample. By comparing the gene expression levels between the treatments and the CK, using |log2FC| ≥ 1&Padjust < 0.05 as the screening criteria, differentially expressed genes were screened, and Gene Ontology (GO) function and Kyoto Encyclopedia of Genes and Genomes (KEGG) enrichment analyses were performed.

#### 4.4.3. qRT-PCR

In order to verify the reliability of the transcriptome sequencing data, 9 DEGs related to heat stress and associated with Ca were selected for qRT-PCR validation. The total RNA of the CK- and T-treated groups was reverse-transcribed into cDNA using the PrimeScript II 1st Strand cDNA Synthesis Kit (TaKaRa, Kyoto, Japan), and SYBRPremix Ex TaqTM II (TliRNaseH Plus) (TaKaRa) was used as a fluorescent dye. LightCycle in qRT PCR Experiment ^®^ 480 II real-time fluorescence quantitative PCR instrument (Roche, Basel, Switzerland) was used for analysis, and DN3137 was used as an internal reference gene. We performed three biological replicates of each reaction and calculated the relative expression of the genes using the 2^−ΔΔCt^ algorithm [54]. The specific primers designed using Prime6.0 software are shown in Table S3.

### 4.5. Data Statistics and Analysis

The experimental data were processed using Excel 2010, and analysis of variance and Duncan’s significance test were conducted using SPSS 23.0 software. Principal component analyses were performed on various indicators using SPSS 23.0. Then, the original indicators were converted into new comprehensive indicators for calculation; the calculation formula was as follows: Zj = ∑mBijXi (i = 1, 2 … p, p ≤ m). In the formula, Zj is the comprehensive score value of the jth principal component, Bij is the eigenvector value of the i-th indicator of the jth principal component, and Xi is the measured value of the i-th indicator.

## 5. Conclusions

This study showed that treatment with moderate amounts of calcium chloride effectively protects *D. nobile* plants from the noxious effects of heat. On the basis of physiological and biochemical analyses, the optimal calcium treatment concentration was found by combining membership function and principal component analysis. At the same time, the possible mechanism of *D. nobile*’s tolerance to heat stress was elucidated by biochemical and transcriptomic analyses. The results showed that the appropriate concentrations of CaCl_2_: improved the efficiency of photosynthetic pigment synthesis; maintained the balance of plant reactive oxygen species metabolism; increased the content of osmoregulation substances; and enhanced plant stress tolerance. Based on the comprehensive physiological and biochemical indicators, the 10 mmol/L treatment had the greatest effect in alleviating heat stress. Transcriptomic testing revealed 7013 differentially expressed genes, of which 2719 were upregulated and 294 were downregulated. The upregulation of genes, such as *HSPA1s*, *HSP90A*, and *HSPBP1*, plays a role *D. nobile*’s tolerance to heat stress. The upregulation of the *ATG8* gene suggests that *D. nobile* may utilize autophagy to alleviate the damage caused by heat stress. In addition, MYB, bHLH, WRKY, and NAC transcription factors are also involved in the tolerance of *D. nobile* to heat stress. However, further functional verification is needed to determine how these upregulated or downregulated genes enable *D. nobile* to resist heat stress. This study provides novel insights on the mechanisms underlying heat stress mitigation by exogenous calcium in plants, and provides useful cues for improving the cultivation of *D. nobile*.

## Figures and Tables

**Table 1:** Effect of CaCl_2_ on the growth of *D. nobile* under heat stress.

Treatment	CK	T0	T1	T2	T3	T4
Leaf length (mm)	2.64 ± 0.19a	1.25 ± 0.15c	1.44 ± 0.13c	2.2 ± 0.11b	1.33 ± 0.25c	0.3 ± 0.12d
Leaf width (mm)	1.55 ± 0.05a	0.59 ± 0.1c	0.68 ± 0.09c	1.2 ± 0.07b	0.38 ± 0.06d	0.27 ± 0.06e
Plant height (mm)	4.47 ± 0.21a	3.7 ± 0.27c	3.87 ± 0.16c	4.18 ± 0.23b	2.65 ± 0.25d	2.48 ± 0.26d
Root Fresh Weight (g)	11.71 ± 0.1a	6.95 ± 0.09c	7.1 ± 0.13c	8.75 ± 0.07b	6.47 ± 0.09d	6.0 ± 0.14e
Stem Fresh Weight (g)	64.06 ± 0.09a	43.04 ± 0.09d	46.21 ± 0.12c	48.31 ± 0.06b	40.39 ± 0.09e	28.46 ± 0.1f
Leaf Fresh Weight (g)	26.97 ± 0.1a	11.01 ± 0.11d	24.55 ± 0.07c	24.98 ± 0.14b	10.47 ± 0.07e	9.69 ± 0.07f
Root Dry Weight (g)	3.02 ± 0.08a	1.89 ± 0.1c	1.99 ± 0.12c	2.28 ± 0.08b	1.7 ± 0.06d	1.44 ± 0.1e
Stem Dry Weight (g)	9.27 ± 0.11a	6.05 ± 0.11c	6.07 ± 0.14c	6.96 ± 0.07b	4.63 ± 0.11d	4.57 ± 0.07d
Leaf Dry Weight (g)	4.88 ± 0.11a	3.54 ± 0.08d	3.94 ± 0.08c	4.42 ± 0.08c	3.07 ± 0.12e	3.05 ± 0.12e
Yellow Leaf Rate (%)	1.40 ± 1.63f	58.44 ± 0.65b	38.9 ± 1.04d	27.78 ± 1.03e	40.99 ± 0.73c	74.2 ± 0.6a
Defoliation Rate (%)	2.36 ± 1.66e	22.1 ± 0.91b	15.56 ± 1.19c	10.65 ± 0.9d	33.48 ± 1.09a	16.71 ± 0.78c
Relative water content (%)	92.95 ± 1.75a	61.68 ± 1.65e	79.16 ± 1.71c	86.75 ± 1.51b	74.25 ± 1.77d	43.57 ± 1.32f

Note: Different lowercase letters indicate significant differences among the treatments (*p* < 0.05).

**Table 2:** Effects of CaCl_2_ on photosynthetic pigments of *D. nobile* under heat stress.

Treatment	Chla (mg/g)	Chlb (mg/g)	Chl a+b (mg/g)	Car (mg/g)
CK	0.7 ± 0.01a	0.34 ± 0.01a	1.04 ± 0.01a	2.07 ± 0.01a
T0	0.53 ± 0.01d	0.27 ± 0.01cd	0.69 ± 0.02f	1.74 ± 0.04d
T1	0.58 ± 0.01c	0.28 ± 0.01c	0.85 ± 0.02c	1.83 ± 0.02c
T2	0.61 ± 0.01b	0.3 ± 0.01b	0.92 ± 0.01b	1.95 ± 0.02b
T3	0.54 ± 0.01d	0.27 ± 0.01d	0.8 ± 0.02d	1.72 ± 0.02d
T4	0.44 ± 0.01e	0.25 ± 0.01e	0.76 ± 0.02e	1.57 ± 0.02e

Note: Different lowercase letters indicate significant differences among the treatments (*p* < 0.05).

**Table 3:** Effect of CaCl_2_ on Comprehensive index value Y (x), weight, membership function value u (x), D value of *D. nobile* under heat stress.

Treatment	Score of Comprehensive Index			Membership Function Value			D Value	Sorting
	Y1	Y2	Y3	U1	U2	U3		
T0	−2.113	−1.807	−1.070	0.179	0.131	0.134	0.163	5
T1	0.183	−2.473	0.230	0.427	0.000	0.557	0.333	3
T2	5.507	0.257	0.720	1.000	0.540	0.718	0.861	1
T3	0.200	2.587	−1.480	0.429	1.000	0.000	0.530	2
T4	−3.780	1.430	1.593	0.000	0.770	1.000	0.275	4
Equal weighting				0.667	0.244	0.087		

## Data Availability

Not applicable.

## Supplementary Materials

The supporting information can be downloaded at: https://www.mdpi.com/article/10.3390/ijms241914692/s1.

## References

[1] Zhang B., Hu Y., Niu Z., Li C., Ou J., Xue Q., Liu W., Chen J., Ding X. (2022). Diversity Evaluation of Dendrobium nobile Germplasm Resources Based on Phenotypic Traits. China Biotechnol..

[2] Zhang S., Tu H., Zhu J., Liang A., Huo P., Shan K., He J., Zhao M., Chen X., Lei X. (2020). *Dendrobium nobile* Lindl. polysaccharides improve follicular development in PCOS rats. Int. J. Biol. Macromol..

[3] Zhao Y., Xing J., Li L., Zhan M., Li F., Lin L., Wang L., Yang W. (2023). Optimization of PEG 200 extraction process and in vitro antioxidant activity of polyphenols from Dendrobium nobile flowers. Sci. Technol. Food Ind..

[4] Zhang Y.W., Shi Y.C., Zhang S.B. (2023). Metabolic and transcriptomic analyses elucidate a novel insight into the network for biosynthesis of carbohydrate and secondary metabolites in the stems of a medicinal orchid *Dendrobium nobile*. Plant Divers..

[5] Liu B., Guo M., Li F., Shi J.S. (2023). *Dendrobium nobile* Lindl. alkaloids (DNLA) inhibits d-galactose-induced hippocampal neuronal senescence through the SIRT1-FoxO1-autophagy axis. Pharmacol. Res.-Mod. Chin. Med..

[6] Cai W., Zhang H., Liu P., Sun Z., Wei X. (2013). Study on the hypoglycemic effect of water extract from *Dendrobium nobile* on streptozotocin induced hyperglycemia in mice. Acta Chin. Med. Pharmacol..

[7] Liang J.S., Deng Y.C., Yu G.C., Gan Y.K. (2012). Anti-fatigue Effects of Polysaccharides Derived from *Dendrobium nobile* Lindl. in Mice. Food Sci..

[8] Hong S., Kim E.Y., Lim S.E., Kim J.H., Sohn Y., Jung H.S. (2022). Dendrobium nobile Lindley Administration Attenuates Atopic Dermatitis-like Lesions by Modulating Immune Cells. Int. J. Mol. Sci..

[9] Wang J.H., Luo J.P., Zha X.Q., Feng B.J. (2010). Comparison of antitumor activities of different polysaccharide fractions from the stems of *Dendrobium nobile* Lindl. Carbohydr. Polym..

[10] Ye G., Zhang J., Xu X., Zeng C., Ye Q., Wang Z. (2023). Comparative Analysis of Water-Soluble Polysaccharides from Dendrobium Second Love ‘Tokimeki’ and Dendrobium nobile in Structure, Antioxidant, and Anti-Tumor Activity In Vitro. Int. J. Mol. Sci..

[11] Wu Y.Q., Si J.P. (2010). Present status and sustainable development of *Dendrobium officinale* industry. Chin. J. Chin. Mater. Med..

[12] Ren Q., Yao Y., Ao Q., Huang F., Xie H. (2023). Study on the meteorological insurance index of high temperature and drought of *Dendrobium nobile* in Chishui City. J. Green Sci. Technol..

[13] Yu Q., Sun W., Han Y., Hao J., Qin X., Liu C. (2022). Exogenous spermidine improves the sucrose metabolism of lettuce to resist high-temperature stress. Plant Growth Regul..

[14] Sarkar S., Aminul Islam A.K.M., Barma N.C.D., Ahmed J.U. (2021). Tolerance mechanisms for breeding wheat against heat stress: A review. S. Afr. J. Bot..

[15] Liu Y., Xi M., Li Y., Cheng Z., Kong F. (2021). Improvement in salt tolerance of iris pseudacorus l. in constructed wetland by exogenous application of salicylic acid and calcium chloride. J. Environ. Manag..

[16] Ghosh S., Bheri M., Pandey G.K. (2021). Delineating Calcium Signaling Machinery in Plants: Tapping the Potential through Functional Genomics. Curr. Genom..

[17] Liang C., Zhang Y., Ren X. (2021). Calcium regulates antioxidative isozyme activity for enhancing rice adaption to acid rain stress. Plant Sci..

[18] Tan W., Meng Q.W., Brestic M., Olsovska K., Yang X. (2011). Photosynthesis is improved by exogenous calcium in heat-stressed tobacco plants. J. Plant Physiol..

[19] Guo Y., Liu Y., Zhang Y., Liu J., Gul Z., Guo X.-R., Abozeid A., Tang Z.-H. (2021). Effects of Exogenous Calcium on Adaptive Growth, Photosynthesis, Ion Homeostasis and Phenolics of *Gleditsia sinensis* Lam. Plants under Salt Stress. Agriculture.

[20] Zhao L., Wang P., Chen C., Song G., Tang H. (2017). Effects of Exogenous Ca^2+^ on the Growth, Physiological Characters of Sophora viciifolia Seedlings in Karst Mountain Area under the Drought Stress. J. Nucl. Agric. Sci..

[21] Young M.D., Wakefield M.J., Smyth G.K., Oshlack A. (2010). Gene ontology analysis for RNA-seq: Accounting for selection bias. Genome Biol..

[22] Minoru K., Michihiro A., Susumu G., Masahiro H., Mika H., Masumi I., Toshiaki K., Shuichi K., Shujiro O., Toshiaki T. (2008). KEGG for linking genomes to life and the environment. Nucleic Acids Res..

[23] Lu Q., Wang Y., Yang H. (2021). Effect of exogenous calcium on physiological characteristics of salt tolerance in Tartary buckwheat. Biologia.

[24] Wang M., Zhang X., Li Q., Chen X., Li X. (2019). Comparative transcriptome analysis to elucidate the enhanced thermotolerance of tea plants (*Camellia sinensis*) treated with exogenous calcium. Planta.

[25] Yin D., Qi J., Deng X., Han X. (2018). Effects of Exogenous Calcium on the Metabolic System of *Pinus sylvestris* var. *mongolica* under Drought Stress. J. Shenyang Agric. Univ..

[26] Hu W., Tian S.B., Di Q., Duan S.H., Dai K. (2018). Effects of exogenous calcium on mesophyll cell ultrastructure, gas exchange, and photosystem II in tobacco (*Nicotiana tabacum* Linn.) under drought stress. Photosynthetica.

[27] Zhang F., Liu J., Xing Y., Li B., Zhang Q. (2022). The Influence of Different Exogenous Substances on Physiology and Chlorophyll Fluorescence Parameters of *Trollius chinensis* Seedling under High Temperature Stress. Mol. Plant Breed..

[28] Xie Y., Liu J., Liao X., Li P. (2017). The effect of calcium stress on the physiological and biochemical characteristics of honeysuckle. Jiangsu Agric. Sci..

[29] Xia H., Gao F., Hu L., Lu X., Liang L. (2019). Effects of Melatonin Applicationon Antioxidant Capacityin Kiwifruit Seedings under High Temperature Stress. Acta Bot. Boreali-Occident. Sin..

[30] Du J.H., Fan D.M., Feng X.D. (2021). Effect of CaCl_2_ on the physiological characteristics of eggplant seedlings under high temperature stress. Mol. Plant Breed..

[31] Sangwan V., Orvar B.L., Beyerly J., Hirt H., Dhindsa R.S. (2010). Opposite changes in membrane fluidity mimic cold and heat stress activation of distinct plant MAP kinase pathways. Plant J..

[32] Paupiere M.J., Muller F., Li H.J., Rieu L., Tikunov Y.M., Visser R.G.F., Bovy A.G. (2017). Untargeted metabolomic analysis of tomato pollen development and heat stress response. Plant Reprod..

[33] Tang J.L., Xu H., Yuan P., He K.J., Wang R.C., Bu F.W. (2020). Advance in relationship between heat shock protein 90 s and thermo-tolerance in plants. Shengwu Jishu Tongbao (Biotechnol. Bull.).

[34] Zhou J., Wang J., Yu J.Q., Chen Z. (2014). Role and regulation of autophagy in heat stress responses of tomato plants. Front. Plant.

[35] Zhai Y.F., Guo M., Wang H., Lu J.P., Liu J.H., Zhang C., Gong Z.H., Lu M.H. (2016). Autophagy, a conversed mechanism for protein degradation, responds to heat, and other abiotic stresses in *Capsicum annuum* L.. Front. Plant Sci..

[36] Liu Q., Zhang G.Y., Shinozaki K. (2000). The Plant Mitogen-activated Protein(MAP) Kinase. Acta Bot. Sin..

[37] Huang H.J., Fu S.F., Tai Y.H., Chou W.C., Huang D.D. (2002). Expression of *Oryza sativa* MAP kinase gene is developmentally regulated and stress-responsive. Physiol. Plant.

[38] Yelena K., Chiu W., Tena G. (2000). Functional analysis of oxidative stress-activated mitogen-activated protein kinase cascade in plants. Proc. Natl. Acad. Sci. USA.

[39] Han L.H., Liu C., Zhang W.W., Li F., Deng F.H., Ruan Z.H. (2019). Gene family identification and bioinformatics analysis of heat shock transcription factors (Hsf) in *Dendrobium officinale*. J. South. Agric..

[40] Jaimes F., Montes R.A.C. (2020). The plant MBF1 protein family: A bridge between stress and transcription. J. Exp. Bot..

[41] Xu X.P. (2006). Physical and functional interactions between pathogen-induced Arabidopsis WRKY18,WRKY40, andWRKY60 transcription factors. Plant Cell.

[42] Li S.J., Fu Q.T., Chen L.G., Huang W.D., Yu D.Q. (2011). Arabidopsis thaliana WRKY25,WRKY26,and WRKY33 coordinate induction of plant thermotolerance. Planta.

[43] Sato H., Mizoi J., Tanaka H., Maruyama K., Qin F., Osakabe Y., Morimoto K., Ohori T., Kusakabe K., Nagata M. (2014). Arabidopsis DPB3-1, a DREB2A interactor, specifically enhances heat stress-induced gene expression by forming a heat stress-specific transcriptional complex with NF-Ysubunits. Plant Cell.

[44] Liao C., Zheng Y., Guo Y. (2017). MYB30 transcription factor regulates oxidative and heat stress responses through ANNEXIN-mediated cytosolic calcium signaling in Arabidopsis. New Phytol..

[45] Wang X.J., Liang L.X., Li L.B., Wang T. (2018). Genome-wide analysis of R2R3-MYB transcription factors in *Phalaenopsis equestris*. Linye Kexue Yanjiu (For. Res.).

[46] Liu T., Shi J., Li M., Ye X., Qi H. (2021). Trehalose triggers hydrogen peroxide and nitric oxide to participate in melon seedlings oxidative stress tolerance under cold stress. Environ. Exp. Bot..

[47] Fan Y.J., Yang K.G., Miao R.S., Wang G., Chun Z., Wu S.D., Pu S.R., Luo A.X. (2022). Transcriptome analysis reveals the effects of red and blue light on the physiological and medicinal components of *Dendrobium denneanum*. Ind. Crops Prod..

[48] Fan Y., Li X., Wang G., Ma J., Liu Y., Xu E., Luo A. (2023). Transcriptome analysis reveals the role of polysaccharide biosynthesis in the detoxification of *Dendrobium nobile* under zinc stress. Int. J. Biol. Macromol..

[49] Vuleta A., Jovanović S.M., Tucić B. (2016). Adaptive flexibility of enzymatic antioxidants SOD, APX and CAT to high light stress: The clonal perennial monocot Iris pumila as a study case. Plant Physiol. Biochem..

[50] Fan Y., Ma J., Wang G., Li X., Liu Y., Xu E., Luo A. (2023). Ultrasonic extraction, structural modification and gastric mucosal cells protective activity of a polysaccharide from *Dendrobium denneanum*. Arab. J. Chem..

[51] Fan Y., Xu E., Ma J., Li X., Liu Y., Xu L., Luo A. (2023). Isolation, Structural Characteristics Analysis of a Vigna unguiculata Polysaccharide VUP80-3 and Its Protective Effect on GES-1 Cells In Vitro. Molecules.

[52] Huang Q., Shen Y.X., Zhang C.J., Luo A.X., Fan Y.J. (2014). Contents of polyphenols and flavonoids in *Dendrobium candidum* and their correlation with antioxidant activity. Chin. J. Appl. Environ. Biol..

[53] Fan Y., Xu E., Wang G., He D., Ma J., Liu Y., Li X., Luo A. (2023). Transcriptional and Physiological Analysis Reveal New Insights into the Regulation of Fertilization (N, P, K) on the Growth and Synthesis of Medicinal Components of *Dendrobium denneanum*. Int. J. Mol. Sci..

[54] Livak K.J., Schmittgen T.D. (2001). Analysis of relative gene expression data using real-time quantitative PCR and the 2^−ΔΔCT^ method. Methods.

