# Supplementary material for: Heat Stress Alleviation by Exogenous Calcium in the Orchid Dendrobium nobile Lindl: A Biochemical and Transcriptomic Analysis
Source: Int J Mol Sci. 2023 Sep 28;24(19):14692. doi: 10.3390/ijms241914692 (PMC10572151; doi:10.3390/ijms241914692)
Supplement: Supplementary file 1 [file ijms-24-14692-s001.zip › ijms-2587588-supplementary.pdf]

Table S1 Effect of $CaCl_2$ on factor complex matrix and eigenvector matrix of *D. nobile* under high temperature stress

Number	Index	Innitial eigenvalue			Eigen vector		
		1	2	3	1	2	3
X1	RWC	0.886	-0.207	-0.294	0.271	-0.105	-0.250
X2	REC	-0.692	0.495	0.420	-0.212	0.251	0.357
X3	MDA	-0.456	0.705	-0.492	-0.140	0.357	-0.418
X4	Chla	0.873	-0.389	-0.229	0.268	-0.197	-0.195
X5	Chlb	0.831	-0.432	-0.026	0.255	-0.219	-0.022
X6	Chla+b	0.856	0.058	0.342	0.262	0.029	0.291
X7	Car	0.911	-0.379	-0.111	0.279	-0.192	-0.094
X8	SOD	0.969	-0.136	-0.028	0.297	-0.069	-0.024
X9	POD	0.792	0.316	0.513	0.243	0.160	0.436
X10	CAT	0.729	0.682	-0.016	0.223	0.345	-0.014
X11	Pro	0.873	0.437	0.207	0.268	0.221	0.176
X12	SS	0.485	0.799	0.147	0.149	0.404	0.125
X13	SP	0.658	-0.519	0.205	0.202	-0.263	0.174
X14	ASA	0.948	-0.230	0.183	0.290	-0.116	0.155
X15	GSH	0.926	0.284	-0.163	0.284	0.144	-0.139
X16	Flavonoids	0.554	0.813	-0.059	0.170	0.411	-0.050
X17	Polyphenol	0.758	0.399	-0.513	0.232	0.202	-0.436
	Eigen value	10.65	3.904	1.385			
	Contribution(%)	62.649	22.965	8.146			
	Cumulative contribution(%)	62.649	85.614	93.760			

Table S2 Total RNA sequencing yield statistics of each sample

Sample	Raw reads	Raw bases	Clean reads	Clean bases	Error rate(%)	Q20(%)	Q30(%)	GC content(%)
CK1	44716426	675218	44198958	655960	0.0264	97.53	92.86	45.99
CK2	44594064	673370	43988822	643505	0.0261	97.61	93.14	45.9
CK3	49723516	750825	49186726	728398	0.0255	97.84	93.66	45.82
T1	44457032	6713011	43889566	644999	0.0257	97.76	93.5	46.45
T2	44442822	671086	43889448	648325	0.0257	97.8	93.54	46.26
T3	44387196	670246	43853072	650225	0.0263	97.55	92.97	45.75

Table S3 Primers used in this study for qRT-PCR assay

**Genes_id**	**Primer**	**Sequence**
TRINITY_DN3260	Forward primer	GATCTCCTTAAAGATAAACAAGCCC
	Reverse primer	AAAGAACAATATCACTGACGTCTCC
TRINITY_DN45157	Forward primer	ACTCTTCAGTTCTAGGAAAACCCA
	Reverse primer	GGGTTGTAAGACTGCAACTTCAT
TRINITY_DN14663	Forward primer	AGAGCTCAGATGGAGAGGAATTT
	Reverse primer	GATGTTCAGATAGTTAGCAGCCAG
TRINITY_DN2523	Forward primer	ACTCCCTTTCCTTTTCATCTCC
	Reverse primer	AATGTCTTCTCGCTGCTGTAGTA
TRINITY_DN3317	Forward primer	CAAAGAGCTCATAGTAGGAAACGAG
	Reverse primer	TACCTACATCAGCAGATACAAGTGC
TRINITY_DN440	Forward primer	CTCTCTAATGAGAAAAGGAAGACGA
	Reverse primer	GTCTCTACATTCGAAACCAGTTCTT
TRINITY_DN3350	Forward primer	TATATGGAATCATACCAGAGAAGGG
	Reverse primer	ACGAATCTTGTCAATATCTGGAGAC
TRINITY_DN19125	Forward primer	AAGGCTTCTGTCCAACTCCA
	Reverse primer	CCACATAAGACTCCACCACAAC
TRINITY_DN7111	Forward primer	GAGAGCTGGTACTATCTCAAGGATG
	Reverse primer	GTCACTCCAATCATGAAGAATCC
TRINITY_DN3137	Forward primer	GAGGTAGGGAAATTAGTTATGGGAG
	Reverse primer	AGTAAAGATTGTGGGCTCAATGTAG

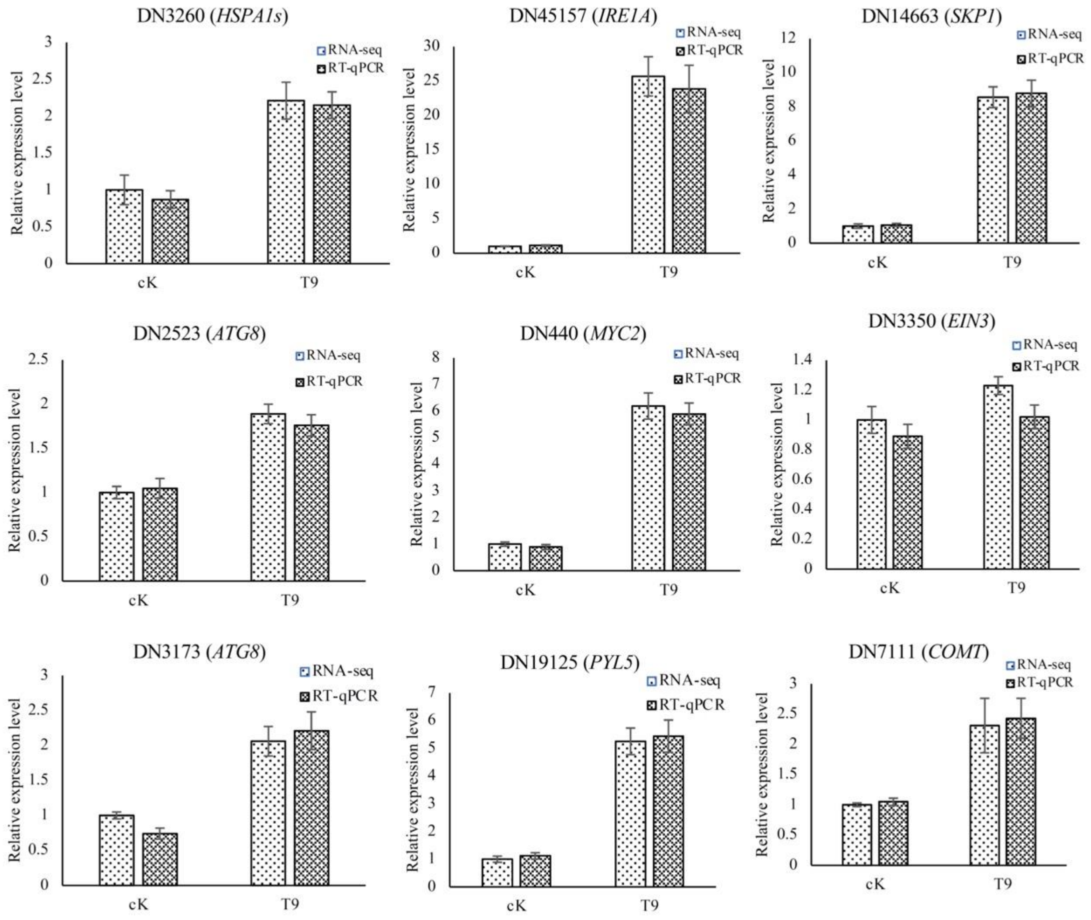

Figure S1. qRT-PCR analysis of differential gene expression levels
